# Draft Genome Sequence of *Acetobacter malorum* CECT 7742, a Strain Isolated from Strawberry Vinegar

**DOI:** 10.1128/genomeA.00620-16

**Published:** 2016-06-23

**Authors:** Florencia Sainz, Albert Mas, María Jesús Torija

**Affiliations:** Biotecnologia Enológica, Department Bioquímica i Biotecnologia, Facultat d‘Enologia. Universitat Rovira i Virgili, Tarragona, Spain

## Abstract

The present article reports the draft genome sequence of the strain *Acetobacter malorum* CECT 7742, an acetic acid bacterium isolated from strawberry vinegar. This species is characterized by the production of d-gluconic acid from d-glucose, which it further metabolizes to keto-d-gluconic acids.

## GENOME ANNOUNCEMENT

*Acetobacter malorum* was proposed as new species in 2002 (1) after a polyphasic study of 34 *Acetobacter* strains. This strain was isolated in rotten apples in Ghent (Belgium) and initially classified as *Acetobacter pasteurianus*. The genus *Acetobacter* is included in the group of acetic acid bacteria (AAB), which are Gram-negative bacteria from the family *Acetobacteraceae*. AAB are aerobic microorganisms that are responsible for vinegar production (2). Its main characteristic is the incomplete oxidation of a wide range of carbohydrates and alcohols yielding the corresponding ketones, aldehydes, and acids that are left in the media (3). The biotechnological industry has taken advantage of this capacity to recover some compounds, such as intermediaries in the production of vitamin C (l-sorbose) and miglitol (antidiabetic drug, after amino-l-sorbose), although these have mostly been applied to members of the genus *Gluconobacter* (4, 5).

After the initial description of *A. malorum*, this species has been found in other environments, generally associated with fruits but also with the processing of these fruits. *A. malorum* has been isolated in rotten grapes from Australia (6), must from healthy grapes from Tarragona, Spain (7), and in fermented grape musts in the Canary Islands (8). *A. malorum* was also isolated from fermented persimmon juices (9) and fermented milk (10). The present strain of *A. malorum* was isolated from strawberry vinegar and has been used as starter culture for the production of different fruit vinegars (11–13). The identification of *A. malorum* is difficult due to the high sequence homology with *Acetobacter cerevisiae* when using the 16S sequence analysis. The use of the internal transcribed spacer (ITS) 16S-23S rRNA coding region has provided conclusive differentiation for both of them (6, 14). The polymorphism in this region has allowed the development of specific TaqMan probes that can be used routinely to differentiate these two species by a culture-independent quantitative PCR technique (15).

The strain *A. malorum* CECT 7742 has provided excellent results in the production of d-gluconic acid from d-glucose without the oxidation of fructose, which has been used for the production of new strawberry beverages based on the presence of fructose as a sweetener and being free of glucose (16).

Genomic DNA was extracted according to the cetyltrimethylammonium bromide (CTAB) method (17). For whole-genome sequencing, the Genome Analyzer Ion Torrent PGM (Thermo Fisher Scientific, Madrid, Spain) was used. Preparation of shotgun libraries was performed according to the protocols of the manufacturers and resulted in 5,149,025 reads (256 bp).

The genome of *A. malorum* CECT 7742 consists of a chromosome with 4.04 Mb and an overall G+C content of 56.78%. The genome was assembled in 331 contigs from 927,367 reads using the software MIRA 4.9.5_2 (18). Prokka (19) was used for automatic annotation and gene detection. The genome harbored 6 rRNA genes, 64 tRNA genes, 3,416 protein-coding genes with predicted functions, and 649 genes coding for hypothetical proteins. Among them, 189 genes encoded dehydrogenases, including membrane PQQ-dependent glucose dehydrogenase and flavin adenine dinucleotide (FAD)-dependent gluconate-2-dehydrogenase and 2-ketogluconate dehydrogenase, responsible for the synthesis of d-gluconic acid and its further oxidation to 2-keto-d-gluconic acid and 2,5diketo-d-gluconic acid, respectively.

### Nucleotide sequence accession numbers.

This whole-genome shotgun project has been deposited at DDBJ/EMBL/GenBank under the accession no. LVHD00000000. The version described in this paper is version LVHD01000000.
